# Supplementary material for: Heparin-based hydrogel scaffolding alters the transcriptomic profile and increases the chemoresistance of MDA-MB-231 triple-negative breast cancer cells
Source: Biomater Sci. 2020 Feb 13;8(10):2786–96. doi: 10.1039/c9bm01481k (PMC7497406; doi:10.1039/c9bm01481k)
Supplement: Supplementary file 2 [file BM-008-C9BM01481K-s002.zip › Supplementary File 4/HP-BhydrogelvControl/Pathways/my_analysis.Gsea.1545201079907/HALLMARK_ADIPOGENESIS.html]

Details for gene set HALLMARK\_ADIPOGENESIS[GSEA]

|  || Dataset | expr.class.cls#HYD\_versus\_CONTROL.class.cls#HYD\_versus\_CONTROL\_repos |
| Phenotype | class.cls#HYD\_versus\_CONTROL\_repos |
| Upregulated in class | CONTROL |
| GeneSet | HALLMARK\_ADIPOGENESIS |
| Enrichment Score (ES) | -0.4371787 |
| Normalized Enrichment Score (NES) | -2.0600104 |
| Nominal p-value | 0.0 |
| FDR q-value | 0.0 |
| FWER p-Value | 0.0 |
Table: GSEA Results Summary

  

Fig 1: Enrichment plot: HALLMARK\_ADIPOGENESIS      
 Profile of the Running ES Score & Positions of GeneSet Members on the Rank Ordered List

  

| PROBE | DESCRIPTION (from dataset) | GENE SYMBOL | GENE\_TITLE | RANK IN GENE LIST | RANK METRIC SCORE | RUNNING ES | CORE ENRICHMENT || 1 | PHLDB1 | na |  |  | 356 | 2.558 | -0.0025 | No |
| 2 | ABCB8 | na |  |  | 359 | 2.554 | 0.0136 | No |
| 3 | RREB1 | na |  |  | 454 | 2.408 | 0.0239 | No |
| 4 | DGAT1 | na |  |  | 636 | 2.172 | 0.0282 | No |
| 5 | ESRRA | na |  |  | 749 | 2.075 | 0.0355 | No |
| 6 | SLC19A1 | na |  |  | 752 | 2.073 | 0.0485 | No |
| 7 | LIPE | na |  |  | 1405 | 1.627 | 0.0246 | No |
| 8 | COL15A1 | na |  |  | 1473 | 1.593 | 0.0311 | No |
| 9 | ANGPTL4 | na |  |  | 1665 | 1.505 | 0.0306 | No |
| 10 | STAT5A | na |  |  | 1889 | 1.409 | 0.0279 | No |
| 11 | ADCY6 | na |  |  | 2160 | 1.292 | 0.0219 | No |
| 12 | SULT1A1 | na |  |  | 2186 | 1.280 | 0.0287 | No |
| 13 | SLC25A10 | na |  |  | 2199 | 1.276 | 0.0361 | No |
| 14 | ITGA7 | na |  |  | 2333 | 1.230 | 0.0369 | No |
| 15 | GADD45A | na |  |  | 2424 | 1.200 | 0.0398 | No |
| 16 | MYLK | na |  |  | 2690 | 1.120 | 0.0330 | No |
| 17 | SCARB1 | na |  |  | 2693 | 1.119 | 0.0400 | No |
| 18 | PIM3 | na |  |  | 2754 | 1.100 | 0.0438 | No |
| 19 | NKIRAS1 | na |  |  | 2891 | 1.065 | 0.0434 | No |
| 20 | SLC5A6 | na |  |  | 3383 | 0.943 | 0.0236 | No |
| 21 | AGPAT3 | na |  |  | 3525 | 0.911 | 0.0219 | No |
| 22 | UCK1 | na |  |  | 3590 | 0.895 | 0.0243 | No |
| 23 | FAH | na |  |  | 3746 | 0.859 | 0.0216 | No |
| 24 | COL4A1 | na |  |  | 3868 | 0.840 | 0.0205 | No |
| 25 | LTC4S | na |  |  | 3921 | 0.828 | 0.0230 | No |
| 26 | SLC1A5 | na |  |  | 4248 | 0.762 | 0.0107 | No |
| 27 | DHRS7 | na |  |  | 4798 | 0.659 | -0.0139 | No |
| 28 | GPHN | na |  |  | 5350 | 0.578 | -0.0392 | No |
| 29 | POR | na |  |  | 5382 | 0.572 | -0.0372 | No |
| 30 | PFKL | na |  |  | 5644 | 0.533 | -0.0476 | No |
| 31 | VEGFB | na |  |  | 5954 | 0.493 | -0.0607 | No |
| 32 | CDKN2C | na |  |  | 6116 | 0.469 | -0.0662 | No |
| 33 | CPT2 | na |  |  | 6409 | 0.429 | -0.0788 | No |
| 34 | CHCHD10 | na |  |  | 6446 | 0.423 | -0.0780 | No |
| 35 | BAZ2A | na |  |  | 6657 | 0.399 | -0.0865 | No |
| 36 | ELMOD3 | na |  |  | 6747 | 0.387 | -0.0888 | No |
| 37 | SSPN | na |  |  | 7075 | 0.351 | -0.1037 | No |
| 38 | MGLL | na |  |  | 7102 | 0.348 | -0.1029 | No |
| 39 | ARAF | na |  |  | 7187 | 0.341 | -0.1051 | No |
| 40 | PPP1R15B | na |  |  | 7322 | 0.323 | -0.1101 | No |
| 41 | C3 | na |  |  | 7510 | 0.306 | -0.1180 | No |
| 42 | NMT1 | na |  |  | 7640 | 0.286 | -0.1230 | No |
| 43 | SORBS1 | na |  |  | 7693 | 0.284 | -0.1239 | No |
| 44 | IDH3G | na |  |  | 7740 | 0.282 | -0.1246 | No |
| 45 | PREB | na |  |  | 7964 | 0.253 | -0.1347 | No |
| 46 | APOE | na |  |  | 8142 | 0.241 | -0.1424 | No |
| 47 | ETFB | na |  |  | 8173 | 0.236 | -0.1425 | No |
| 48 | CD302 | na |  |  | 8272 | 0.222 | -0.1463 | No |
| 49 | CYC1 | na |  |  | 8715 | 0.179 | -0.1684 | No |
| 50 | ACADS | na |  |  | 8847 | 0.159 | -0.1742 | No |
| 51 | PFKFB3 | na |  |  | 8864 | 0.157 | -0.1741 | No |
| 52 | ITSN1 | na |  |  | 9112 | 0.139 | -0.1862 | No |
| 53 | PEX14 | na |  |  | 9195 | 0.126 | -0.1897 | No |
| 54 | DDT | na |  |  | 9271 | 0.121 | -0.1929 | No |
| 55 | SLC27A1 | na |  |  | 9282 | 0.120 | -0.1926 | No |
| 56 | COQ3 | na |  |  | 9331 | 0.112 | -0.1945 | No |
| 57 | AIFM1 | na |  |  | 9365 | 0.109 | -0.1955 | No |
| 58 | ME1 | na |  |  | 9632 | 0.084 | -0.2089 | No |
| 59 | ECH1 | na |  |  | 9762 | 0.069 | -0.2153 | No |
| 60 | AK2 | na |  |  | 9863 | 0.056 | -0.2202 | No |
| 61 | DHCR7 | na |  |  | 10032 | 0.036 | -0.2288 | No |
| 62 | ABCA1 | na |  |  | 10121 | 0.026 | -0.2332 | No |
| 63 | BCL6 | na |  |  | 10150 | 0.022 | -0.2346 | No |
| 64 | RETSAT | na |  |  | 10420 | 0.005 | -0.2487 | No |
| 65 | QDPR | na |  |  | 10490 | -0.003 | -0.2523 | No |
| 66 | OMD | na |  |  | 10750 | -0.032 | -0.2657 | No |
| 67 | CRAT | na |  |  | 11449 | -0.107 | -0.3017 | No |
| 68 | HIBCH | na |  |  | 11525 | -0.116 | -0.3049 | No |
| 69 | DHRS7B | na |  |  | 11598 | -0.126 | -0.3079 | No |
| 70 | PTCD3 | na |  |  | 11722 | -0.141 | -0.3135 | No |
| 71 | TKT | na |  |  | 11730 | -0.142 | -0.3130 | No |
| 72 | FZD4 | na |  |  | 11894 | -0.162 | -0.3205 | No |
| 73 | MAP4K3 | na |  |  | 12278 | -0.210 | -0.3393 | No |
| 74 | SPARCL1 | na |  |  | 12400 | -0.222 | -0.3442 | No |
| 75 | PPM1B | na |  |  | 12604 | -0.234 | -0.3534 | No |
| 76 | ACLY | na |  |  | 12653 | -0.241 | -0.3544 | No |
| 77 | ACO2 | na |  |  | 12768 | -0.255 | -0.3588 | No |
| 78 | CD151 | na |  |  | 12935 | -0.276 | -0.3658 | No |
| 79 | SLC25A1 | na |  |  | 13006 | -0.284 | -0.3677 | No |
| 80 | DBT | na |  |  | 13141 | -0.305 | -0.3728 | No |
| 81 | COX8A | na |  |  | 13216 | -0.312 | -0.3747 | No |
| 82 | ESYT1 | na |  |  | 13486 | -0.351 | -0.3866 | No |
| 83 | PEMT | na |  |  | 13524 | -0.357 | -0.3863 | No |
| 84 | ACADL | na |  |  | 13643 | -0.378 | -0.3901 | No |
| 85 | GPAM | na |  |  | 13645 | -0.378 | -0.3877 | No |
| 86 | UCP2 | na |  |  | 13665 | -0.379 | -0.3863 | No |
| 87 | LAMA4 | na |  |  | 13738 | -0.393 | -0.3876 | No |
| 88 | REEP6 | na |  |  | 13830 | -0.411 | -0.3898 | No |
| 89 | BCL2L13 | na |  |  | 14064 | -0.426 | -0.3994 | No |
| 90 | GRPEL1 | na |  |  | 14110 | -0.430 | -0.3990 | No |
| 91 | PPARG | na |  |  | 14161 | -0.436 | -0.3989 | No |
| 92 | TST | na |  |  | 14230 | -0.448 | -0.3996 | No |
| 93 | GPX4 | na |  |  | 14284 | -0.460 | -0.3995 | No |
| 94 | CCNG2 | na |  |  | 14302 | -0.464 | -0.3974 | No |
| 95 | RAB34 | na |  |  | 14411 | -0.478 | -0.4001 | No |
| 96 | NDUFA5 | na |  |  | 14435 | -0.481 | -0.3982 | No |
| 97 | CS | na |  |  | 14588 | -0.504 | -0.4030 | No |
| 98 | ACOX1 | na |  |  | 14591 | -0.504 | -0.3999 | No |
| 99 | HADH | na |  |  | 14717 | -0.526 | -0.4032 | No |
| 100 | GPX3 | na |  |  | 14790 | -0.536 | -0.4035 | No |
| 101 | LPL | na |  |  | 14944 | -0.553 | -0.4081 | No |
| 102 | NDUFS3 | na |  |  | 14957 | -0.555 | -0.4052 | No |
| 103 | PQLC3 | na |  |  | 15048 | -0.571 | -0.4063 | No |
| 104 | ALDOA | na |  |  | 15303 | -0.618 | -0.4157 | No |
| 105 | UQCR11 | na |  |  | 15707 | -0.683 | -0.4326 | No |
| 106 | LPCAT3 | na |  |  | 15716 | -0.685 | -0.4287 | No |
| 107 | MCCC1 | na |  |  | 15879 | -0.720 | -0.4326 | Yes |
| 108 | COQ9 | na |  |  | 15892 | -0.722 | -0.4287 | Yes |
| 109 | STOM | na |  |  | 15937 | -0.731 | -0.4263 | Yes |
| 110 | ALDH2 | na |  |  | 15979 | -0.740 | -0.4238 | Yes |
| 111 | GPD2 | na |  |  | 16014 | -0.750 | -0.4208 | Yes |
| 112 | DNAJC15 | na |  |  | 16119 | -0.772 | -0.4214 | Yes |
| 113 | ECHS1 | na |  |  | 16197 | -0.787 | -0.4205 | Yes |
| 114 | TANK | na |  |  | 16289 | -0.808 | -0.4201 | Yes |
| 115 | ATP1B3 | na |  |  | 16327 | -0.816 | -0.4169 | Yes |
| 116 | DLD | na |  |  | 16423 | -0.837 | -0.4166 | Yes |
| 117 | ACADM | na |  |  | 16495 | -0.853 | -0.4149 | Yes |
| 118 | DECR1 | na |  |  | 16501 | -0.855 | -0.4097 | Yes |
| 119 | PGM1 | na |  |  | 16509 | -0.856 | -0.4047 | Yes |
| 120 | DLAT | na |  |  | 16528 | -0.860 | -0.4002 | Yes |
| 121 | ATL2 | na |  |  | 16696 | -0.897 | -0.4033 | Yes |
| 122 | APLP2 | na |  |  | 16744 | -0.912 | -0.3999 | Yes |
| 123 | SCP2 | na |  |  | 16821 | -0.932 | -0.3980 | Yes |
| 124 | ADIPOR2 | na |  |  | 16920 | -0.962 | -0.3971 | Yes |
| 125 | DRAM2 | na |  |  | 16934 | -0.965 | -0.3916 | Yes |
| 126 | UBQLN1 | na |  |  | 16987 | -0.983 | -0.3881 | Yes |
| 127 | UQCRC1 | na |  |  | 17067 | -1.006 | -0.3859 | Yes |
| 128 | IDH3A | na |  |  | 17098 | -1.014 | -0.3811 | Yes |
| 129 | ARL4A | na |  |  | 17123 | -1.023 | -0.3758 | Yes |
| 130 | SNCG | na |  |  | 17129 | -1.025 | -0.3696 | Yes |
| 131 | TALDO1 | na |  |  | 17146 | -1.029 | -0.3639 | Yes |
| 132 | RNF11 | na |  |  | 17262 | -1.070 | -0.3632 | Yes |
| 133 | PHYH | na |  |  | 17292 | -1.081 | -0.3578 | Yes |
| 134 | DNAJB9 | na |  |  | 17375 | -1.111 | -0.3551 | Yes |
| 135 | NDUFB7 | na |  |  | 17428 | -1.125 | -0.3507 | Yes |
| 136 | PLIN2 | na |  |  | 17478 | -1.144 | -0.3460 | Yes |
| 137 | SAMM50 | na |  |  | 17513 | -1.160 | -0.3404 | Yes |
| 138 | CMBL | na |  |  | 17518 | -1.162 | -0.3333 | Yes |
| 139 | MDH2 | na |  |  | 17620 | -1.207 | -0.3309 | Yes |
| 140 | ELOVL6 | na |  |  | 17624 | -1.209 | -0.3234 | Yes |
| 141 | CHUK | na |  |  | 17628 | -1.210 | -0.3159 | Yes |
| 142 | IMMT | na |  |  | 17677 | -1.232 | -0.3106 | Yes |
| 143 | NDUFAB1 | na |  |  | 17728 | -1.254 | -0.3053 | Yes |
| 144 | YWHAG | na |  |  | 17839 | -1.303 | -0.3028 | Yes |
| 145 | MTCH2 | na |  |  | 17852 | -1.311 | -0.2951 | Yes |
| 146 | SDHC | na |  |  | 17944 | -1.344 | -0.2914 | Yes |
| 147 | TOB1 | na |  |  | 17952 | -1.348 | -0.2832 | Yes |
| 148 | RTN3 | na |  |  | 18037 | -1.405 | -0.2787 | Yes |
| 149 | RIOK3 | na |  |  | 18045 | -1.411 | -0.2701 | Yes |
| 150 | ANGPT1 | na |  |  | 18126 | -1.457 | -0.2651 | Yes |
| 151 | ENPP2 | na |  |  | 18138 | -1.460 | -0.2564 | Yes |
| 152 | IDH1 | na |  |  | 18161 | -1.475 | -0.2482 | Yes |
| 153 | PRDX3 | na |  |  | 18171 | -1.480 | -0.2393 | Yes |
| 154 | MGST3 | na |  |  | 18186 | -1.490 | -0.2306 | Yes |
| 155 | MRPL15 | na |  |  | 18190 | -1.490 | -0.2213 | Yes |
| 156 | UQCR10 | na |  |  | 18208 | -1.505 | -0.2126 | Yes |
| 157 | UQCRQ | na |  |  | 18242 | -1.522 | -0.2047 | Yes |
| 158 | CMPK1 | na |  |  | 18294 | -1.560 | -0.1975 | Yes |
| 159 | JAGN1 | na |  |  | 18350 | -1.604 | -0.1902 | Yes |
| 160 | IFNGR1 | na |  |  | 18491 | -1.701 | -0.1868 | Yes |
| 161 | PDCD4 | na |  |  | 18523 | -1.724 | -0.1775 | Yes |
| 162 | G3BP2 | na |  |  | 18622 | -1.808 | -0.1712 | Yes |
| 163 | GBE1 | na |  |  | 18637 | -1.827 | -0.1603 | Yes |
| 164 | GHITM | na |  |  | 18722 | -1.917 | -0.1526 | Yes |
| 165 | SDHB | na |  |  | 18744 | -1.946 | -0.1414 | Yes |
| 166 | ATP5O | na |  |  | 18790 | -2.007 | -0.1310 | Yes |
| 167 | SOD1 | na |  |  | 18799 | -2.015 | -0.1186 | Yes |
| 168 | LIFR | na |  |  | 18803 | -2.023 | -0.1060 | Yes |
| 169 | REEP5 | na |  |  | 18817 | -2.045 | -0.0937 | Yes |
| 170 | COQ5 | na |  |  | 18825 | -2.050 | -0.0810 | Yes |
| 171 | ACAA2 | na |  |  | 18837 | -2.070 | -0.0685 | Yes |
| 172 | COX6A1 | na |  |  | 18964 | -2.287 | -0.0606 | Yes |
| 173 | SUCLG1 | na |  |  | 19027 | -2.431 | -0.0485 | Yes |
| 174 | CAT | na |  |  | 19052 | -2.529 | -0.0337 | Yes |
| 175 | HSPB8 | na |  |  | 19137 | -3.038 | -0.0188 | Yes |
| 176 | COX7B | na |  |  | 19184 | -3.465 | 0.0007 | Yes |
Table: GSEA details [plain text format]

  

Fig 2: HALLMARK\_ADIPOGENESIS      
 Blue-Pink O' Gram in the Space of the Analyzed GeneSet

  

Fig 3: HALLMARK\_ADIPOGENESIS: Random ES distribution      
 Gene set null distribution of ES for **HALLMARK\_ADIPOGENESIS**
